# Cigarette smoke condensate attenuates phorbol ester-mediated neutrophil extracellular trap formation

**DOI:** 10.4314/ahs.v17i3.33

**Published:** 2017-09

**Authors:** Refilwe Philadelphia Bokaba, Ronald Anderson, Annette Johanna Theron, Gregory Ronald Tintinger

**Affiliations:** 1 Department of Immunology, Faculty of Health Sciences, University of Pretoria, Pretoria, South Africa; 2 Institute for Cellular and Molecular Medicine, SAMRC Extramural Unit for Stem Cell Research and Therapy, Department of Immunology, Faculty of Health Sciences, University of Pretoria, Pretoria, South Africa; 3 Tshwane Academic Division of the National Health Laboratory Service, Pretoria South Africa; 4 Department of Internal Medicine, University of Pretoria and Steve Biko Academic Hospital, Pretoria, South Africa

**Keywords:** Neutrophils, reactive oxygen species, respiratory infection, smoking

## Abstract

**Background:**

Neutrophil extracellular traps (NETs) constitute a network of chromatin fibres containing histone and antimicrobial peptides that are released by activated neutrophils. NETs protect the host against infection by trapping and facilitating phagocytosis of potentially harmful pathogens.

**Objectives:**

The aim of the current study was to investigate the effects of cigarette smoke condensate (CSC) on phorbol-ester (PMA)-mediated NETosis in vitro.

**Methods:**

Isolated human blood neutrophils were exposed to PMA (6.25 ng/ml) in the presence or absence of CSC (40–80 µg/ml) for 90 min at 37oC. NET formation was measured using a spectrofluorimetric procedure to detect extracellular DNA and fluorescence microscopy was used to visualize nets. Oxygen consumption by PMA-activated neutrophils was measured using an oxygen sensitive electrode.

**Results:**

Activation of neutrophils with PMA was associated with induction of NETosis that was significantly attenuated in the presence of CSC (40 and 80 µg/ml), with mean fluorescence intensities of 65% and 66% of that observed with untreated cells, respectively, and confirmed by fluorescence microscopy. The rate and magnitude of oxygen consumption by activated neutrophils pre-treated with CSC (80 µg/ml) was significantly less than that observed with untreated cells (73% of the control system), indicative of decreased production of reactive oxidants in the presence of CSC.

**Conclusion:**

The inhibition of NETosis observed in the presence of CSC correlated with attenuation of oxygen consumption by PMA-activated neutrophils suggesting a mechanistic relationship between these events. If operative in vivo, smoking-related attenuation of NETosis may impair host immune responses and increase the risk of respiratory infections.

## Introduction

Neutrophil extracellular trap formation (NETosis) is a recently described host defence mechanism characterised by the decondensation of chromatin and nuclear segmentation resulting in the extracellular release of netlike structures^1^–^3^. NET formation can be triggered by a number of physiological and non-physiological activators such as interleukin-8 (IL-8), lipopolysaccharide (LPS), phorbol esters, interferon-γ (IFN-γ), various types of bacteria and their products, viruses, fungi and the complement cleavage component, C5a. The phorbol ester, phorbol-12-myristate-13-acetate (PMA) activates NETosis in vitro^4^,^5^. PMA-induced NET formation requires protein kinase C (PKC)-mediated activation of the membrane-associated electron-transport, superoxide-generating complex, NADPH oxidase, as well as the activation of the enzyme peptidyl arginine deiminase 4 (PAD4) which leads to the citrullination of histones^6^. This is followed by the release of decondensed chromatin web-like fibres entangled with citrullinated histones and impregnated with anti-microbial peptides and proteins including neutrophil elastase and myeloperoxidase^3^,^7^–^10^. Activation of NADPH oxidase is apparently a critical requirement for NETosis since patients with chronic granulomatous disease (CGD) who are unable to generate reactive oxidants, do not produce NETs when activated with PMA. Furthermore, diphenyleneiodonium chloride, an inhibitor of NADPH-oxidase activity, when added to neutrophils effectively attenuates PMA-mediated NET formation^11^,^12^. NET formation protects the host against infection which is achieved via the capture of microbes and viruses in the net-like structures of decondensed chromatin fibres which are impregnated with anti-microbial agents resulting in the immobilisation, localisation and possibly elimination of the infection^10^,^12^. In this setting, NET formation is potentially an advantageous host defence mechanism.

Cigarette smoking is a common lifestyle habit worldwide, with 21% of the population older than 15 years, smoking tobacco on a regular basis^13^. Cigarette smoke is composed of chemical compounds that include tar, nicotine, carbon monoxide, polycyclic aromatic hydrocarbons and high concentrations of oxidants and free radicals^14^. Cigarette smoke has been shown to increase oxidant production by neutrophils^14^,^15^, and to promote oxidative processes such as lipid peroxidation, protein carbonylation, thiol peroxidation and DNA oxidation. Cigarette smoking also recruits macrophages and neutrophils to the lungs and increases the citrullination of proteins^16^–^20^.

To our knowledge, there are no publications to date evaluating the effects of cigarette smoke and cigarette smoke condensate on NETosis. In the current study, we investigated the effects of exposure of isolated human blood neutrophils to cigarette smoke condensate (a surrogate for cigarette smoke) on PMA-induced NET formation in vitro.

## Methodology

### Ethics approval

The study was approved by the Research Ethics Committee of the Faculty of Health Sciences, University of Pretoria (Approval No. 2015/358).

### Participants

The participants were healthy non-smoking individuals who were not taking any medication. They were aged 19–49 years old and consisted of 5 males and 7 females (general characteristics displayed in Table 1). Measurement of phorbol 12-myristate 13-acetate activated NETosis in the absence and presence of CSC (40–80 µg/ml) using spectrofluorimetry was performed on neutrophils from all 12 donors, while confirmatory microscopy, oxygen consumption and viability were performed on cells from 5, 6 and 9 donors respectively.

**Table 1:** Demographic data of the 12 healthy non-smoking donors involved in the study.

Characteristics	Current Non-smokers (n=12)
**Age (Mean, year range):**	28(19–49)
**Gender:** Female Male	7 5
**Race:** Black White	2 10

### Reagents and Chemicals

Alexa Fluor 488-labeled goat anti-rabbit secondary antibody, 5 mM Sytox orange and 4′,6-diamidino-2-phenylindole (DAPI) were all purchased from Life Technologies (Pty) Ltd (USA). Histopaque-1077 was purchased from Sigma Aldrich (Pty) Ltd (Johannesburg, South Africa) and the polyclonal rabbit anti-histone H4 (citrulline 3) was purchased from Merck Millipore (Pty) Ltd (Eastern Cape, South Africa). Propidium iodide (50 µg/ml DNA Prep-Stain) was purchased from Beckman Coulter Pty (Ltd) (Johannesburg, South Africa) and the cigarette smoke condensate (CSC) from Murty Pharmaceuticals Inc, Lexington (KY) and dissolved in dimethyl sulphoxide to give a stock concentration of 40mg/ml. The total amount of condensate generated during the combustion of one cigarette is about 26.3 mg, therefore the concentrations of the condensate used in this study were relevant in the context of the smoking habit^21^. The CSC was prepared by burning University of Kentucky's 1R3E standard cigarettes and extracting the total particulate matter generated into DMSO using a smoking machine 22–23. The constituents of CSC include a complex mixture of phenolic compounds such as phenols, cresols and dihydroxybenzenes, as well as hydrogen cyanide, acrolein and formaldehyde^24^–^25^. Unless indicated the remaining chemicals and reagents mentioned in the methods were purchased from Sigma-Aldrich.

## Methods

### Neutrophil isolation

Following to obtaining informed consent, neutrophils were prepared from heparinised (5 units of preservative-free heparin/ml) venous blood and separated from mononuclear leukocytes by centrifugation on Histopaque-1077 cushions at 754 x g for 25 minutes at room temperature. The resultant cell pellet was suspended in phosphate-buffered saline (PBS, 0.15M, pH 7.4) and sedimented with 3% gelatine to remove most of the erythrocytes. After centrifugation, erythrocytes were removed by selective lysis with 0.84% ammonium chloride at 4°C for 10 minutes. Following centrifugation (355 x g for 10min), the supernatant fluid was removed and the cells were washed in PBS and the neutrophils were then re-suspended to 1x10^7^/ml in PBS and held on ice until used. Purity and viability were assessed using flow cytometry.

### Exposure of neutrophils to PMA and CSC

A total of 4x10^6^ of cells (0.4 ml) was added to 3.6 ml of Hanks' balanced salt solution (HBSS, pH 7.4, indicator-free) and incubated for 5 minutes at 37°C. Following incubation the cells were exposed to either DMSO (solvent control, 2µl/ml) or CSC (40–80 µg/ml, final). Following incubation for 10 minutes at 37°C, the cells were activated by the addition of PMA (6.25 ng/ml), a potent activator of NETosis, and incubated for 90 minutes at 37°C. Thereafter, the tubes were vortexed and centrifuged for 5 minutes, 4°C at 355 x g to pellet the neutrophils. Three ml of the supernatant was then pipetted into new 5 ml tubes and the pellet resuspended and the cells retained for analysis of viability.

### Spectrofluorimetry

Three microlitres (3 µl) of 5 mM Sytox orange, a DNA-reactive, fluorescent dye, was added to the harvested supernatant fluids in reaction cuvettes and transferred to the cuvette holder of a Hitachi 650-10S fluorimeter with the excitation and emission wavelengths set at 530 nm and 590 nm respectively. Fluorescence intensity as an index of NETosis was recorded as metered fluorescence units (MFUs).

### Fluorescence Microscopy

Microscopy was performed to confirm that the extracellular DNA demonstrated by other techniques was the product of NETosis. Neutrophil suspension (1.25x10^5^ cells in 250 µl) was allowed to adhere to glass cover slips for 30 minutes. PMA (6.25 ng/ml) or an equal volume of HBSS in the presence and absence of CSC (80µg/ml) was added to the adherent cells and the coverslips were then incubated for 120 minutes at 37°C 5% CO_2_. The cells were then fixed with 4% paraformaldehyde for 10 minutes, washed 3 times in PBS and blocked with HBSS containing 5% goat serum and 5% bovine serum albumin for 30 minutes at 37°C. Neutrophils were incubated overnight with polyclonal rabbit anti-histone H4 (citrulline 3, Merck Millipore) and washed three times in PBS. Following addition of Alexa Fluor 488-labeled goat anti-rabbit secondary antibody, DNA present in the specimen was stained with the DNA-binding dye, DAPI, for 2 minutes and analysed for NET formation by fluorescence microscopy using a Zeiss Axio Vert. A1 fluorescence microscope and Axion Vision Software (Zeiss, Johannesburg, South Africa). Representative photomicrographs were taken, and used for the generation of counts of cells undergoing NETosis by a single non-blinded observer. Results are expressed as percentage of NET forming cells.

### Cell Viability

This was measured after a 90 minutes incubation following the addition of PMA to neutrophils using a flow cytometric propidium idodide-based dye exclusion assay. The cells (4x10^6^/ml) were incubated for 5 minutes with propidium iodide (50 µg/ml) DNA Prep-Stain, and cell viability assessed flow cytometrically with the results expressed as percentage of viable cells.

### Oxygen consumption

A total of 4 x10^6^ of cells (0.4 ml) were added to 1.6 ml of HBSS and incubated for 10 minutes at 37°C in the absence or presence of CSC (80 µg/ml). The cells were then transferred to the thermoregulated compartment of an Oxygen Electrode (Hansatech, Instruments Ltd., Norflok, UK). After a stable baseline was reached (in about 1 minute) the cells were stimulated with PMA (6.25 ng/ml) and oxygen consumption measured over a 10 min period and recorded as relative units.

### Statistical analysis

Statistical significance was calculated from raw data collected from each series of experiments using a Mann-Whitney non-parametric test for analysis of the experiments measuring the effects of CSC on PMA-induced NET formation, oxygen consumption and cell viability. A computer-based software system was used (Graph Pad Instat 3®)) and the results expressed as the median of the corresponding CSC-free control systems with percentile values for each series of experiments.

## Results

### Effects of CSC on NET formation

#### Spectrofluorimetric analysis

**Figure 1:**
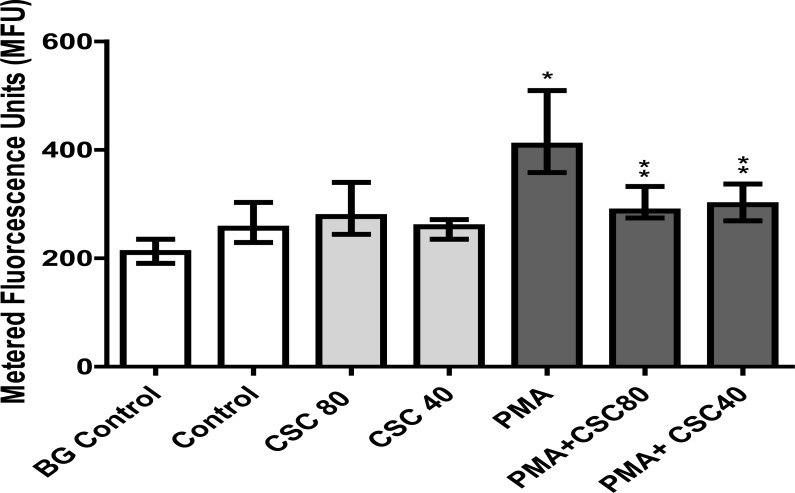
The effects of CSC (40 and 80 µg/ml) on PMA (6.25 ng/ml)-activated NETosis using spectrofluorimetric analysis for DNA measurement with results expressed as the median values in metered fluorescence units (MFUs) and with 25% and 75% percentiles. NETosis was significantly increased in PMA-activated neutrophils (*P<0.0002), while in the presence of CSC (40 and 80 µg/ml), the magnitude of PMA-induced NETosis was significantly attenuated (**P<0.0005 and **P<0.0001), respectively.

These are shown in Figure 1 which depict the effects of activation of neutrophils with PMA (6.25 ng/ml) on NET formation following 90 minutes of incubation at 37°C which resulted in a statistically significant increase in NET formation (P<0.0002). Figure 1 also depicts the effects of CSC (40 and 80 µg/ml) on PMA-activated NETosis. Exposure of PMA-activated cells to CSC resulted in attenuation of NETosis , the values for systems treated with 40 and 80 µg/ml being 65% and 66% of the corresponding untreated control system respectively (P<0.0001 and P<0.0005).

#### Microscopic analysis

**Figure 2:**
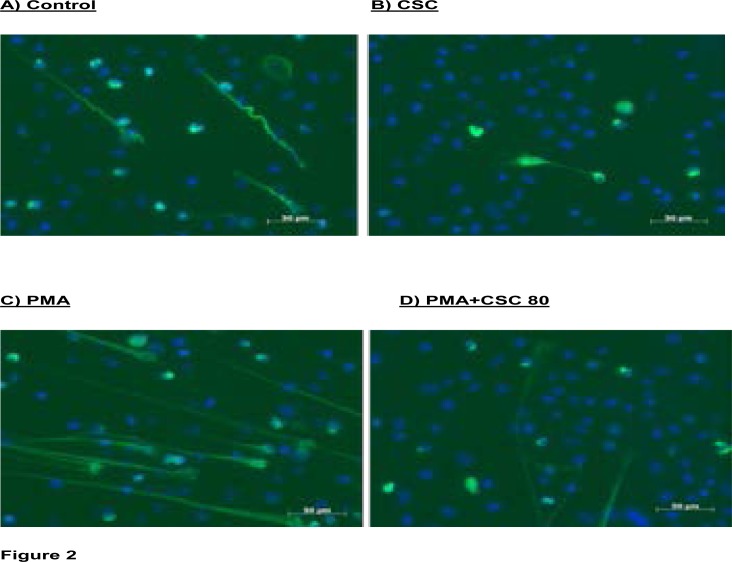
Fluorescence microscopic images from a single representative experiment (5 in the series) showing NETosis of resting neutrophils in the absence (A) and presence (B) of CSC (80 µg/ml) following 120 minutes incubation, as well as NETosis following activation of the cells with PMA (6.25 ng/ml) in the absence (C) and presence (D) of CSC.

**Figure 3:**
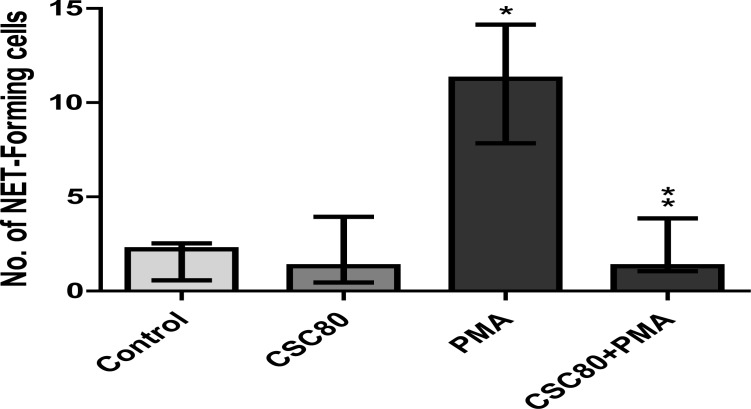
NET formation by resting and PMA (6.25 ng/ml)-activated neutrophils in the absence and presence of CSC (80 µg/ml) measured microscopically according to the numbers of NET-forming cells. The results of 5 separate experiments are expressed as the median of NET-forming cells for each system with percentile values of 25% and 75%. PMA induced NETosis was significant (*P<0.0001) and attenuation of NETosis in PMA-activated systems treated with CSC (80 µg/ml) obtained statistical significance (**P<0.0001) as indicated

These results are shown in Figures 2 and 3, which show representative fluorescence micrographs depicting PMA-activated NETosis in the absence and presence of CSC at a concentration of 80 µg/ml (Figure 2). Averaged data from a larger number of experiments (Figure 3) resulted in statistical significance (P<0.0001) on the effects of neutrophils activated with PMA (6.25 ng/ml) on NET formation and further confirming the inhibitory effects of exposure of neutrophils to CSC on PMA-activated NETosis (P<0.0001).

#### Viability analysis

**Figure 4:**
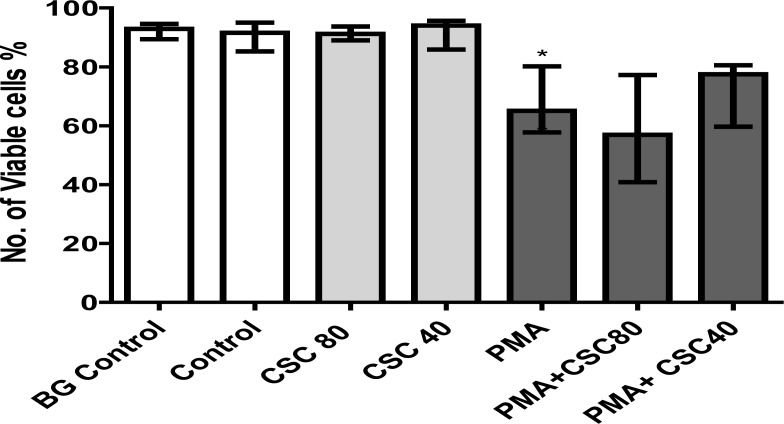
The effects of exposure of unstimulated and PMA (6.25 ng/ml)-activated neutrophils to CSC (40-80 µg/ml) following 90 minutes incubation on neutrophil viability. The results of 9 separate experiments are expressed as the median valueswith percentile values of 25% and 75%. PMA (6.25 ng/ml) activation resulted in (*P<0.0003) loss of viability.

These results are presented in Figure 4 which show the viabilities of unstimulated and PMA-activated neutrophils in the absence and presence of CSC at 40 and 80 µg/ml following 90 minutes incubation at 37 °C. Exposure of the cells to CSC alone did not affect viability, while activation with PMA resulted in statistically significant (P<0.0003) loss of viability consistent with lytic NETosis, with no additive effects of CSC.

#### Oxygen consumption

**Figure 5:**
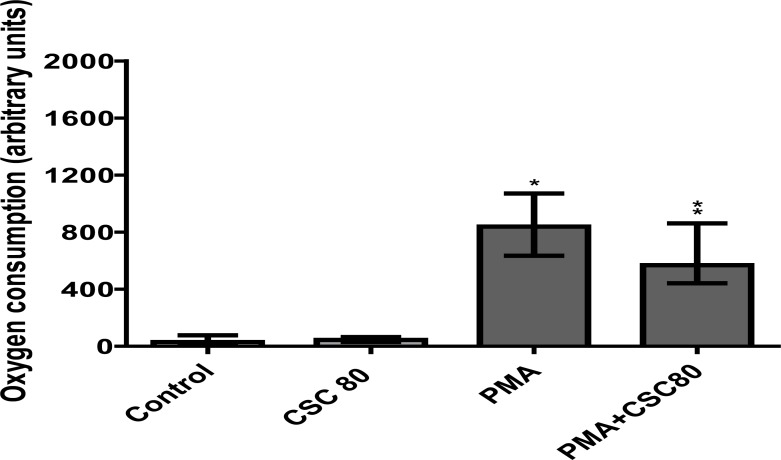
The effects of CSC (80 µg/ml) on oxygen consumption measured over a 10 minute period by unstimulated and PMA (6.25 ng/ml)-activated neutrophils. The results of 6 separate experiments are expressed as median arbitrary units. PMA-induced NET formation of neutrophils obtained statistical significance as indicated (*P<0.0001). **P<0.0001 for comparison of the PMA-activated systems in the presence and absence of CSC.

**Figure 6:**
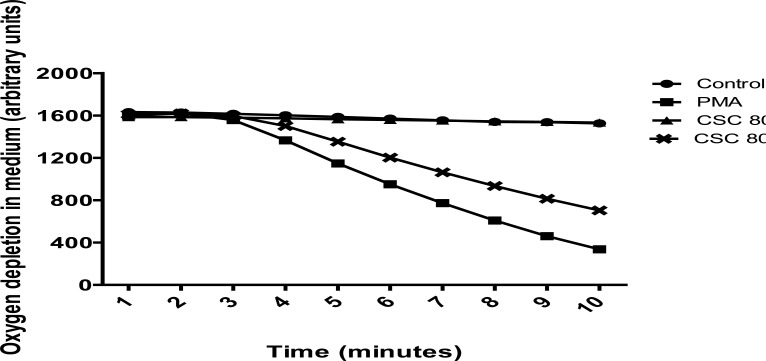
Time course of oxygen depletion in medium by unstimulated and PMA (6.25 ng/ml)-activated neutrophils in the absence and presence of CSC (80 µg/ml). The data of 6 separate experiments are averaged for each time point.

The results presented in Figure 5 are those for the averaged total amounts of oxygen consumed over the 10 minute incubation period by resting, unstimulated neutrophils, as well as by PMA (6.25 ng/ml)-activated cells in the absence and presence of CSC (80 µg/ml). They demonstrate firstly, that exposure of neutrophils to PMA (6.25 ng/ml) results in marked consumption of oxygen by the cells (P<0.0001), and, secondly, that exposure of PMA-activated, but not resting cells, to CSC, results in statistically significant attenuation of oxygen consumption (73% of the CSC-free control system, P<0.0001). The time course of these experiments, averaged for each experimental system (resting and PMA-activated neutrophils in the absence and presence of CSC) are shown in Figure 6.

## Discussion

The current study was designed to investigate the effects of cigarette smoke condensate (CSC) on phorbol ester-mediated neutrophil extracellular trap formation (NETosis) in vitro. The phorbol ester, PMA, activates the neutrophil membrane-associated electron-transporting enzyme, NADPH-oxidase, which generates reactive oxidant species (ROS) from molecular oxygen^26^. ROS, in turn, activates intracellular signaling pathways which induce NETosis^11^. During NETosis, decondensation of nuclear chromatin precedes the disintegration of nuclear membranes and mixing of nucleic acids with cytosolic granules to form vacuoles^1^. The contents of these vacuoles are released into the extracellular environment forming web-like structures that trap microbial pathogens and promote their destruction by proteases and ROS^1^,^8^.

In the current study, PMA-mediated NETosis was detected 90 minutes following addition of the stimulant using both a spectrofluorimetric procedure and fluorescence microscopy. In the presence of CSC, significant dose-dependent attenuation of NETosis was observed and this was associated with a reduction in the rate and magnitude of oxygen consumption by PMA-activated neutrophils. In this setting, the rate and magnitude of oxygen consumption by activated neutrophils reflects the activity of the ROS-generating NADPH oxidase. CSC has been reported to inhibit the activity of NAPDH oxidase^27^. Importantly, CSC did not affect the viability of neutrophils. As ROS have been shown to play an important role in inducing NETosis, it is likely that the inhibitory effects of CSC on NETosis may be attributed to attenuation of ROS production by neutrophils. Interestingly, nicotine, a constituent of CSC has recently been reported to activate NETosis^28^. A possible explanation for this discrepancy is that CSC and cigarette smoke contain many other substances in addition to nicotine which may exert differential effects on NADPH oxidase and NETosis. The inhibitory effects of CSC on neutrophil NADPH oxidase activity and NETosis appears to contradict the well-recognized pro-oxidative potential of cigarette smoke in the lungs of smokers. However, additional mechanisms may contribute to smoking-induced oxidative stress in the setting of decreased neutrophil NADPH oxidase activity. These include recruitment of macrophages and T-cells to the lungs, release of pro-inflammatory cytokines by lymphocytes and airway epithelial cells, enhanced release of matrix metalloproteinases (MMPs) and other proteases by neutrophils, as well as priming of neutrophils in the lungs with increased spontaneous release of O_2_- and H_2_O_2_^29^–^30^.

Although the experimental design of the current study focused primarily on the effects of CSC on neutrophils from non-smokers, a limited series of experiments were performed to evaluate the effects of cigarette smoke (CS) on NETosis by comparing individuals who smoke with those who do not smoke. The magnitude of PMA-mediated NETosis was significantly decreased in smokers compared to non-smokers with a similar reduction in the rate and magnitude of oxygen consumption by PMA-activated neutrophils (data not shown).

The inhibitory effects of CSC on NETosis observed in the current study may be clinically relevant as NETosis promotes the destruction of microbial pathogens by facilitating phagocytosis and proteolytic degradation of microbes ensnared by extracellular NETs. Therefore, exposure to cigarette smoke may impair the immune response of the host and predispose these individuals to infections such as those caused by *Streptococcus pneumoniae, Mycobacterium tuberculosis* and the influenza virus^31^. Indeed, it is well recognized that smokers have an increased risk of colonization by *Streptococcus pneumoniae* and are more likely to develop invasive pneumococcal disease^32^. Cigarette smoking has also been reported to alter the immunological response to pulmonary tuberculosis (PTB) increasing susceptibility to PTB^33^–^34^ and delaying the clearance of acid-fast bacilli from the sputum of patients started on anti-tuberculous chemotherapy^35^. In addition, smoking may be associated with treatment failure and higher recurrence rates compared to non-smokers treated for PTB^31^. PTB and pneumococcal *pneumonia* are highly prevalent conditions in Africa accounting for significant morbidity and mortality.

## Conclusion

The results of the current study suggest that CSC attenuates PMA-mediated NETosis in vitro. If operative in the clinical setting, this effect may at least in part explain the exaggerated risk of infection, including *pneumococcal* disease, observed in individuals who smoke.
